# Natural killer cells in the central nervous system

**DOI:** 10.1186/s12964-023-01324-9

**Published:** 2023-11-29

**Authors:** Zhiyuan Ning, Ying Liu, Daji Guo, Wei-Jye Lin, Yamei Tang

**Affiliations:** 1 Department of Neurology, Sun Yat-Sen Memorial Hospital, Sun Yat-Sen University, Guangzhou, 510120 China; 2 Brain Research Center, Sun Yat-Sen Memorial Hospital, Sun Yat-Sen University, Guangzhou, 510120 China; 3 Guangdong Provincial Key Laboratory of Malignant Tumor Epigenetics and Gene Regulation, Guangdong-Hong Kong Joint Laboratory for RNA Medicine, Medical Research Center, Sun Yat-Sen Memorial Hospital, Sun Yat-Sen University, Guangzhou, 510120 China; 4 Guangdong Province Key Laboratory of Brain Function and Disease, Zhongshan School of Medicine, Sun Yat-Sen University, Guangzhou, 510080 China; 5 Nanhai Translational Innovation Center of Precision Immunology, Sun Yat-Sen Memorial Hospital, Foshan, 528200 China

**Keywords:** NK cells, Central nervous system, Multiple sclerosis, Aging, Neurodegenerative diseases

## Abstract

Natural killer (NK) cells are essential components of the innate lymphoid cell family that work as both cytotoxic effectors and immune regulators. Accumulating evidence points to interactions between NK cells and the central nervous system (CNS). Here, we review the basic knowledge of NK cell biology and recent advances in their roles in the healthy CNS and pathological conditions, with a focus on normal aging, CNS autoimmune diseases, neurodegenerative diseases, cerebrovascular diseases, and CNS infections. We highlight the crosstalk between NK cells and diverse cell types in the CNS and the potential value of NK cells as novel therapeutic targets for CNS diseases.

Video Abstract

**Supplementary Information:**

The online version contains supplementary material available at 10.1186/s12964-023-01324-9.

## Background

NK cells, belonging to the innate lymphoid cell (ILC) family, are crucial for innate immune responses, participating in pathogen defense, immunosurveillance, and homeostasis maintenance [1]. Unlike T and B cells expressing diverse rearranged antigen receptors, NK cells express a spectrum of complex germline-encoded immune receptors to distinguish normal and abnormal cells. The balance between activating and inhibitory signaling inputs determines the outcome of NK cell activation or tolerance. NK cells are activated in an antigen-unspecific mode, rendering themselves as an ideal candidate for rapid immune responses.

Conventional wisdom has it that the central nervous system (CNS) is an immune-privileged site since the blood–brain barrier (BBB) and blood-cerebrospinal fluid barrier (BCSFB) insulate peripheral immune cells from entering the CNS. However, new perspectives in meningeal lymphatic vessels [2] and dural sinuses [3] have dramatically altered this viewpoint and expanded our understanding of CNS immune surveillance: constant and intimate interactions between CNS and peripheral immune system play a critical role in maintaining homeostasis [4]. NK cells seem to be forgotten but important immune cells communicating with the CNS: they can act as the bridge in the crosstalk between immune system and CNS in the context of normal aging and many neurological diseases, such as CNS autoimmune diseases (i.e., multiple sclerosis), neurodegenerative diseases (i.e., Alzheimer’s disease), cerebrovascular diseases (i.e., stroke), and infections. In this review, we describe the biological functions of NK cells and their involvement in the homeostatic and diseased states of CNS, and the therapeutic potential of NK cells-targeting strategies.

### NK cell biology

#### Features and classifications

NK cells originate from the same ancestor as T and B lymphocytes—common lymphocyte progenitor (CLP) and mature in the bone marrow and lymphoid organs [5, 6]. The IFN-γ production and cytolytic capabilities are widely acknowledged as functional hallmarks of mature NK cells [7]. In terms of phenotypes, expression of CD56 and lack of CD3 (CD56^+^CD3^−^) are typical features of human NK cells, while in murine NK cells, which notably lack CD56 expression, the prevalent markers include NK1.1, NKp46, or CD49b (DX5 antigen) [8]. On the basis of adhesion molecule CD56 and Fcγ receptor CD16 expression, there are two classical subsets of human circulating NK cells: CD16^+^ CD56^dim^ and CD16^−^ CD56^bright^ populations respectively [1]. CD56^bright^ NK cells are generally considered as the immature phenotype and further differentiate into CD56^dim^ counterparts [9]. The former constitute a small part in the circulation endowed with the capacity to produce numerous cytokines, while the latter represent the majority of NK cells in the peripheral blood and exert powerful cytotoxicity [1, 10]. Apart from peripheral blood NK cells, tissue-resident NK (trNK) cells are also the important component of the NK cell pool, which respond to the complex and flexible microenvironment. They express distinct surface receptors, shared CD16^−^ CD56^bright^ phenotype in different human tissues [7, 11]. Although murine type 1 innate lymphoid cells (ILC1s) have been considered as the counterpart of human trNK cells based on their similarity in surface markers and functions, this borderland between ILC1s and trNK cells is still a point of current controversy in the field [9, 12, 13].

#### Molecular mechanism of NK cells activation and functions

Contrarily to T and B lymphocytes, NK cells do not undergo the somatic gene rearrangement but express a diverse repertoire of activating, co-stimulatory and inhibitory NK cell receptors (NKRs). The combination of signals received from these receptors determines whether NK cells are activated or restrained [6]. Generally, normal cells constitutively express various ligands for NK-inhibitory receptors to restrain NK cells and maintain self-tolerance [14]. These constant communications assist in the functional maturation of NK cells, which is termed “licensing” or “education” [15]. Once unhealthy cells lose NK-inhibitory ligands, “licensed” NK cells can be rapidly activated with minimal stimulus, which refers to “missing self” hypothesis [16]. The activation of NK cells also relies on the enhanced activating signals, which is known as “induced self” hypothesis [17]. Activating NKRs, attached with adapter proteins containing immunoreceptor tyrosine-based activation motifs (ITAMs), bind to “induced-self” ligand especially under pathological circumstances such as tumorigenesis, infectious states, and DNA damage, thereby triggering a cascade of activation signals [17]. The threshold of NK cells activation is also determined by cytokine exposure, especially interleukin (IL)-2 and IL-15 [18]. IL-2 is predominantly generated by activated CD4^+^ T cells, while IL-15 is produced by monocytes, macrophages, and dendritic cells in the periphery and neurons and glia cells in the brain. These two cytokines trigger similar downstream signaling pathways essential for NK cell survival, proliferation, priming and effector functions [6, 19–23]. Furthermore, other cytokines like IL-12, IL-18, and IL-27 also contribute to the preactivated state of NK cells, while TGFβ and IL-10 assist in the development and maintenance of tolerant phenotypes [24, 25]. Another major mechanism known for NK cell recognition and activation, aside from the “missing self” and “induced self” hypotheses, is the Antibody-Dependent Cellular Cytotoxicity (ADCC). This mechanism is initiated by the binding of CD16, the most potent activating receptor on NK cells, to the Fc domain of an IgG antibody [1, 26]. Upon stimulation, NK cells can efficiently kill target cells via multiple mechanisms including secretion of granules-containing perforin and granzymes, and upregulation of FAS ligand (FASL) and TNF-related apoptosis-inducing ligand (TRAIL) [1, 6]. In addition to cytotoxic function, NK cells can also produce various cytokines and chemokines, such as pro-inflammatory factors like IFN-γ, tumor necrosis factor (TNF)-α, and the growth factor granulocyte–macrophage colony-stimulating factor (GM-CSF) [8, 18].

### NK cells in the CNS

**Fig. 1:**
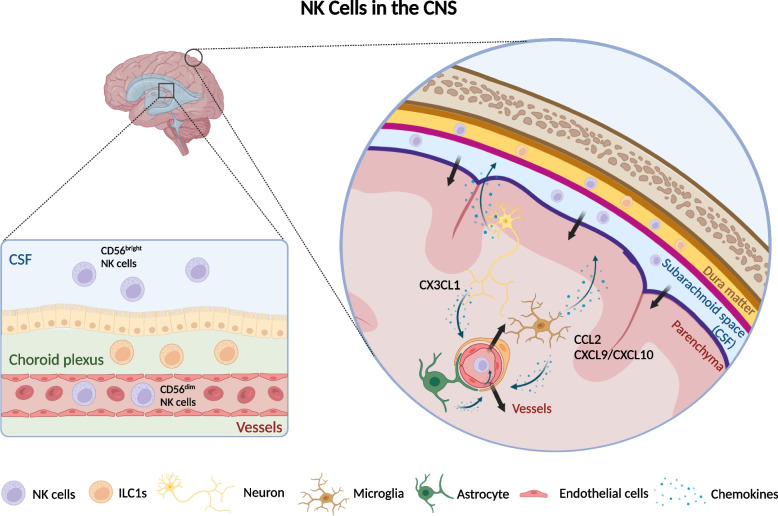
NK cells in the CNS. Conventional NK cells are divided into CD56^dim^ and CD56^bright^ groups. The CD56^dim^ cells predominantly circulate in the peripheral blood, whereas the CD56^bright^ subset chiefly populates the CSF. In the steady state of CNS, cNK cells are localized within the meninges while tissue-resident NK cells or ILC1s reside in the choroid plexus and meninges. In disease states, brain-resident cells, like neurons, microglia, astrocytes and endothelia cells, are able to release different chemokines to guide NK cells in their infiltration into the brain parenchyma. (Created with BioRender.com)

NK cells are ubiquitously distributed across diverse tissues and organs of human body [27]. Although it’s hard to obtain brain tissue samples from healthy people, mass cytometry and single-cell transcriptomic analyses confirm the existence of NK cells in the healthy mouse brain, which make up a small fraction (1.1 ± 0.14%) of the total immune cells in CNS compartments and reside mostly in the boundaries, like meninges and choroid plexus, instead of parenchyma [28, 29]. With reference to molecular features, they exhibit remarkably higher levels of IL-2R and CD27, equivalently to human CD56^bright^ subgroups [28, 30]. In human, most NK cells in blood are CD56^dim^ while the CD56^bright^ population represents the majority in cerebrospinal fluid (CSF), perhaps because CD56^bright^ NK cells possess more adhesive and migratory capabilities related to passage through the BBB at steady state [31, 32]. However, both of them notably increase during neuroinflammation, indicating that CD56^dim^ NK cells can be recruited in diseases (Fig. 1) [32].

How NK cells reach the CNS has been poorly investigated. A higher degree of NK cell migration to the CNS seems to occur in the condition of the BBB breakdown [33]. This complex process of NK cell trafficking requires multifaceted regulation, especially the aid of adhesion and chemokine networks. Blocking the interaction between the cell adhesion molecule VLA-4 on NK cells and VCAM-1 on endothelial cells precludes the ingress of NK cells into the brain parenchyma [34]. As to chemotaxis, NK cells express a variety of receptors in response to chemokines and migrate toward a chemokine gradient [35]. CX3CR1, highly expressed by CD56^dim^ subsets, binds to neuron-derived CX3CL1, which guides the NK cell recruitment in different situations, such as ischemic stroke, parasitic infection, and experimental autoimmune encephalomyelitis (EAE) [36–39]. Beyond that, glial and vascular cells also secrete chemokines, like CCL2, CXCL10, and CXCL12, which mediate the chemoattraction of NK cells to the brain [40–43]. Interestingly, as recent evidence suggests skull bone marrow cavities can be an independent source of myeloid cells and B cells, the possibility might exist that NK cells in the CNS directly derive from their precursor cells in the skull [44, 45].

The meningeal lymphatic vessels offer a drainage system allowing the egress of both molecules and immune cells from the subarachnoid space into the deep cervical lymph nodes (dCLNs) as well as the superficial cervical lymph nodes (sCLNs), establishing a link between the peripheral immune system and the CNS [46]. Consistent with this, human CD56^bright^ NK cells preferentially express CCR7 and CD62L but express CXCR1, CXCR2, and CX3CR1 at a very low degree, which is crucial for the entry of NK cells into lymph nodes [47]. It is possible that these CD56^bright^ NK cells in the CSF may patrol the CNS, support normal brain functions by secreting cytokines and finally exit via meningeal lymphatic vessels under physiological conditions. The damage to meningeal lymphatics occurs during aging and various neurological disorders, possibly leading to an unbalanced meningeal immunity and the consequential impairment of NK cell functions [46].

Typically, conventional NK cells (cNK) refer to those circulating in peripheral blood and are divided to CD56^dim^ and CD56^bright^ groups as mentioned above. Recent studies have broadened this perspective, coining the terms “tissue-resident NK cells” or “non-conventional NK cells” by identifying two distinct phenotypes of liver NK cells in mice, namely CD49a^−^DX5^+^ cNK and CD49a^+^DX5^−^ trNK cells, as well as human liver-resident NK cells with different features, which holds a new era in the research of trNK cell biology [12, 48–50]. Subsequently, these cells have been identified and characterized in an array of organs beyond the liver, including the lungs, uterus, and intestines in both humans and mice [12, 51–53]. TrNK cells share functional similarities with CD56^bright^ cNK cells owing to the robust capacities of cytokine production and weak cytotoxicity, but there are distinct features to distinguish them. For instance, high expression of adhesion molecules makes trNK cells preserve within tissues and restrain their egress into the circulation [7]. Murine NK cells and ILC1s exhibit different developmental trajectories, indicating a distinct lineage of trNK cells in the ILC family [54]. According to the latest research, there are three subtypes of NK1.1^+^ cells in the healthy murine CNS, including CD49a^−^DX5^+^ cNK cells, CD49a^+^DX5^+^ intILC1s, and CD49a^+^DX5^−^ ILC1s. The ILC1s account for over half of immature CD27^+^CD11b^−^ NK cells and locate predominately in the choroid plexus and meninges. Besides CD49a, they also constitutively express other ILC1 markers, like CD200R, adhesion molecules, such as lectin CD69, chemokine receptors, including CXCR3 and CXCR6, and the death ligand TRAIL. Moreover, they depend on T-bet instead of Eomes [55]. Considering the similar molecular features between CNS ILC1s and trNK cells, these data indicate the existence of CNS-resident NK cells, which collectively contribute to a subgroup of the NK cell lineage different from cNK cells [12]. They might function as a gatekeeper in the CNS allowing immune cell infiltration and initiating inflammation because of their locations and IFN-γ production [56]. It is valuable to provide the evidence of trNK cells existing in the human CNS, discriminate them from cNK cells in molecular and functional features especially related to the CNS, which offers more precise and efficient targets of regulating neuroimmune interactions.

### NK cells in CNS diseases

#### Autoimmunity

**Fig. 2:**
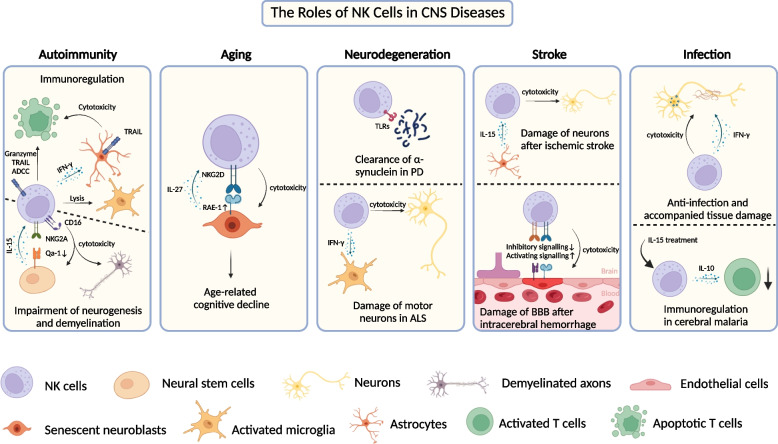
The roles of NK cells in CNS diseases. In EAE, NK cells play a pivotal role in curtailing excessive autoimmune responses by eliminating autoreactive T cells and activated microglia, and fostering anti-inflammatory astrocytes. The malfunction of NK cells in their immunoregulatory roles may accelerate the progression of the disease. Conversely, NK cells also lyse neural stem cells and participate in demyelination, resulting in brain damage. During normal aging, NK cells accumulate in aged dentate gyrus, where they engage in elimination of senescent neuroblasts, consequently impairing neurogenesis. The roles of NK cells in neurodegenerative diseases remain elusive. NK cells may serve as scavengers of α-synuclein aggregates in PD, but concurrently exacerbate ALS by causing neuronal damage and creating inflammatory milieu. During stroke onset, NK cells infiltrate into brain parenchyma and kill hypoxic neurons and endothelia cells, thereby aggravating secondary brain injury. In CNS infectious diseases, NK cells exert positive effects in combating infections, but their enhanced cytotoxicity and cytokine production may yield adverse consequences. Treatment of cerebral malaria with IL-15 successfully improves outcomes of the disease by activating NK cells to modulate deleterious responses of pathogenic T cells. (Created with BioRender.com)

Multiple Sclerosis(MS) is a chronic autoimmune disease of the CNS, mainly characterized by inflammation, demyelination and neurodegeneration [57]. Even though the exact etiology and mechanism are heterogeneous, attacks of peripheral immune cells on the brain and spinal cord are fundamental to lesions formation of MS, especially autoreactive T cells [58]. A number of reports have demonstrated that protective NK cells inhibit T cell-mediated tissue damage in MS and strategies involved in promoting their regulatory capacity contribute to remission of the disease [59–63]. However, other studies suggested that NK cells could exacerbate the pathology by inducing demyelination and impairing neurogenesis [64, 65]. Therefore, their roles in the pathophysiology of CNS autoimmunity are still uncertain and contradictory (Fig. 2).

By co-culturing NK cells with autologous T cells isolated from peripheral blood, researchers found CD56^bright^ NK cells could recognize activated T cells instead of resting T cells via several receptors, such as NKp30, NKp44, and NKG2D, and killed them by transferring granzyme A (GrA) and GrK or through TRAIL-dependent cytotoxicity [59, 60, 66]. Degranulation of GrK induces mitochondrial malfunction and excess ROS production in activated T cells, resulting in their death [67, 68]. GrK-expressing CD56^bright^ NK cells are enriched in both periventricular regions and demyelinating lesions of MS individuals. These cells exhibit GrK polarization towards T cells, corroborating their capacities to infiltrate the CNS via the choroid plexus and suppress neuroinflammation in vivo [31, 69]. Apart from CD56^bright^ subsets, the CD56^dim^ NK cells from MS patients show cytotoxicity against both resting and activated T cells via ADCC [63].

Compromised regulatory effects due to both defective NK cell function and enhanced resistance of T cells may accelerate the pathology [59]. For instance, downregulation of DNAM-1 on NK cells leads to their reduced responses, while increased expression of HLA-E and decreased expression of CD155 on patient-derived T cells result in reduced sensitivity to NK cell cytotoxicity, causing out-of-control autoimmunity [31, 59]. In addition, recent research identified a CD8 positive NK cell subtype (CD8^+^NK) correlated with lower risk of relapse, which might be attributed to its negative regulation of CD4^+^ T cells. Activated CD4^+^ T cells upregulate the expression of HLA-G to inhibit NK cells, while CD8^+^NK cells express decreased HLA-G receptors (ILT2 and KIR2DL4) and help themselves escape from suppression [70]. Hence, NK cells limit CNS autoimmunity to a certain extent by interacting with autoreactive T cells. Disruption in this regulatory mechanism could potentially exacerbate the disease.

Different experiments on EAE models also point to their role in restricting the inflammatory process since ablation of NK cells or limitation of their trafficking to the CNS exacerbates the disease while expansion of NK cells by IL­2 complexes lessens the disease severity [36, 61]. In addition to direct interactions between NK cells and T cells, NK cells can play a protective role by indirectly altering the T cell response during EAE. For example, NK cells are capable of killing activated microglia to suppress the polarization of myelin-reactive Th17 cells [61]. Moreover, meningeal NK cells produce IFN-γ to drive an anti-inflammatory phenotype of astrocytes, which can induce T cell apoptosis via TRAIL-dependent mechanism [62]. Applying laquinimod, an immunomodulator as an oral treatment for MS, to EAE models activates NK cells and upregulates the expression of CD226, which weakens MHC-II-mediated antigen presentation of CD155^+^ dendritic cells and therefore suppress the activation of T cells [71, 72]. In addition to regulating T cells, CD56^bright^ NK cells from patients’ blood exhibit upregulated cholinergic system, which is associated with regulation of immune system [73]. Transferring these choline acetyltransferase (ChAT)-expressing NK cells to EAE models significantly improve the disease outcome possibly by downregulating Qa-1 expression on proinflammatory CCR2^+^Ly6C^hi^ monocytes [39].

The immunoregulative role of NK cells are enhanced after some MS therapies. Interferon β(IFN-β) is a well-known disease-modifying therapy designed for relapsing remitting MS(RRMS), the possible mechanism of which includes the increased number of CD56^bright^ NK cells after treatment [74, 75]. Daclizumab is a therapeutic humanized monoclonal antibody targeting CD25 to abolish high-affinity IL2-R, which was once approved for the treatment of adult MS owing to the promising clinical effects but has been withdrawn nowadays due to its severe adverse events [68, 76]. IL-2 is first defined as a “T cell growth factor” as its potent role in T cell expansion and daclizumab is designed to block the activation and expansion of T cells to limit autoimmunity [77]. However, there is a remarkable expansion of CD56^bright^ NK cells accompanied by a significant reduction in the ratios of CD4^+^ T/NK and CD8^+^ T/NK cells in both the CSF and peripheral blood of patients administered with daclizumab [63, 78]. That’s likely because CD56^bright^ NK cells express great abundance of CD122 with intermediate IL-2 affinity and bind excess IL-2 after CD25 blockade [74, 79]. Additionally, a differentiation switch of ILCs from lymphoid tissue inducer (LTi) cells to NK cell lineage occurs after applying daclizumab, contributing to enlarging NK cells [80]. Moreover, daclizumab also restores compromised regulatory functions of NK cells by enhancing the expression of GrA and GrK, as well as augmenting CD155 expression on CD4^+^ T cells [31, 60]. NK cells might also be involved in the adverse events (AEs) of daclizumab. For example, in a clinical study of 31 patients, the incidence of skin AEs was 77%, with 19% of participants suffering moderate to severe skin rash and 13% terminating treatment. Notably, there was a robust infiltration of CD56^+^ cells in the skin of patients with moderate and severe rashes [81]. Even though the occurrence and the clinical severity of skin AEs did not correlate with the expansion of CD56^bright^ NK cells in PBMCs, this phenomenon might indicate that the overreaction of CD56^bright^ NK cells could drive autoimmunity in other tissues and cause untoward reactions [81, 82]. Another study reported a daclizumab-treated patient developed CNS vasculitis and found the decreasing number of regulatory CD4 T cells and lack of the expansion of CD56^bright^ NK cells [83]. The possible explanation of this participant might be that unresponsive CD56^bright^ NK cells could not compensate for the daclizumab-mediated Tregs depletion, leading to the excessive immune response [84]. Therefore, we hypothesize that the inappropriate response of CD56^bright^ NK cells might be one of the mechanisms by which AEs occur, which needs further exploration. Alemtuzumab is another approved monoclonal antibody for relapsing MS, which acts by binding to CD52 expressed on T and B cells, resulting in depletion of CD52 positive T and B cells followed by subsequent repopulation [85, 86]. Unexpectedly, amplified CD56^bright^ NK cells are observed after treatment, which persist for a long duration, at least 2 years, indicating their indispensable roles in disease modification [87–89]. Autologous hematopoietic stem cell transplantation (HSCT) is an emerging strategy for patients with aggressive MS, achieving the reestablishment of immune tolerance and a long-duration remission [90]. After receiving HSCT, increased CD56^bright^ NK cells replace the old CD56^dim^ subset and regulate detrimental Th1 and Th17 cells via the NKG2D-dependent cytotoxicity [91–93].

Paradoxically, while various studies have pointed to the protective role of NK cell, some work concludes opposite views. For instance, NK cells are found to accumulate near vascular areas in the brain sections of MS patients. With the assistance of activated T cells, these NK cells penetrate the demyelinated cortical gray matter and ultimately cause perivascular cortical demyelination via ADCC. Intriguingly, depletion of NK cells ameliorates perivascular lesions instead of subpial damage, suggesting their trafficking to the brain through vascular routes. These facts indicate CD56^dim^ NK cells may be responsible for this harmful demyelination [64]. Another study characterized a NK-mediated impairment of neurogenesis in MS. There are two main neurogenic niches in the adult brain, the subgranular zone (SGZ) in the dentate gyrus and the subventricular zone (SVZ) along the lateral ventricles, where neural stem cells (NSCs) maintain self-renewal and differentiate into neurons and glia [94]. NK cells are detected within the SVZ region of MS patients and persist even during the chronic inflammatory phase under the auspices of IL-15 derived from NSCs. NSCs downregulate the expression of Qa1, transform into missing-self cells and become the targets of NK cells, which results in the impaired reparative capacities during the recovery phase of neuroinflammation [65]. The plausible explanations for these contradictory observations may encompass variations in the disease’s distinct phases, the discrete subsets of NK cells, the disparate microenvironments where NK cells accumulate, and the divergent target cells engaging with NK cells. The contradictory results underscore the intricate interplay between immune cells and the CNS environment, while also illuminating a promising role of NK cells in regulating the CNS immunity.

Regarding the roles of ILC1s, the expression of chemokine receptors CXCR3 and CXCR6 of ILC1s is decreased during EAE, which might be attributed to the receptor internalization caused by increasing levels of chemokines exposure, suggesting their recruitment to the inflamed brain [55]. Infiltrated ILC1s are discernible in both the meninges and parenchyma of EAE and facilitate the invasion of encephalitogenic Th17 cells from CNS borders to the parenchyma by producing inflammatory cytokines, suggesting their pathogenic role in CNS autoimmunity [95].

#### Aging

Human aging is a physiological, dynamic and complex process accompanied with time-dependent malfunction at multiple levels [96]. The mechanism underlying age-related cognitive decline has remained obscure. Increasing evidence has suggested the immune system is actively involved in the process of brain aging, not just a passive bystander [97–99].

Cellular senescence is one of the main features of aging [100], referring to stable cell cycle arrest and the consequent profound phenotypic alterations in response to intrinsic and extrinsic stresses [101]. Senescent cells often show dramatic changes in their secretome with a variety of pro-inflammatory cytokines and chemokines, termed the senescence-associated secretory phenotype (SASP) [102]. SASP can be a potent initiating signal to facilitate immune surveillance and clearance by recruiting and activating different components of innate and adaptive immunity [102, 103]. The dentate gyrus serves as the gateway to the hippocampus critical for memory formation and adult neurogenesis [104]. Recent work evaluated the peripheral immune cells in the aged rodent and human dentate gyrus and identified NK cells with the most infiltration and significant changes compared with other lymphocytes and monocytes. Besides, the expansion limited to the dentate gyrus rather than other brain regions indicates the existence of specific microenvironment suitable for NK cells [99]. In this aging neurogenic niche, neuroblasts undergo cellular senescence and display a SASP, which promotes in-situ accumulation and cytotoxicity of NK cells and in turn augments self-clearance of aged neuroblasts [88]. Specifically, senescent neuroblasts produce IL-27 to support NK cell survival and upregulate RAE1, which triggers NK cell “induced-self” recognition pattern and cytolytic function, causing neuroblast death and cognitive deterioration in old age (Fig. 2) [99]. Although removing senescent cells through transgenic methods or senolytic drugs have improved cognitive impairment in aging mice [105, 106], protecting aged neuroblast by NK cell killing significantly improves synaptic plasticity and cognitive function in old mice [99]. This indicates that by clearing senescent cells, it might cause unpredictable consequences owing to the diversity of cellular senescence. Targeting NK cells may serve as a therapeutic strategy to protect impaired neuroblasts and mitigate the age-related cognitive decline. In addition to NK cells, the number of ILC1s increases during aging in the murine CNS, but remains largely uninvestigated [89].

It should be noted that NK cells undergo senescence with age as well. Selective deletion Ercc1 in mouse hematopoietic cells impedes DNA repair pathways and causes premature aging only in the immune system. Interestingly, an aged immune system drives senescent phenotypes in non-lymphoid tissues including the brain, suggesting brain aging might be affected by the dysfunctional immune system [107]. With advanced age, NK cell cytotoxic functions and cytokine-releasing capacities remarkably decrease [108]. One possible hypothesis is deficits in immune surveillance during aging might result in the accumulation of senescent cells and a chronic and low-grade inflammation, which causes tissue damage and age-related diseases [109]. Further investigations need to clarify whether altered NK cell phenotypes and functions during aging affect the CNS and drives the age-related functional declines.

#### Neurodegeneration

Neurodegenerative disorders are age-related CNS diseases, usually generally characterized by deposition of misfolded protein aggregates and progressive loss of neurons [110]. Both innate and adaptive immune responses are suggested to be involved in the progression of neurodegeneration, as revealed by the emerging evidences from clinical studies and animal models [111, 112].

##### Alzheimer’s disease

Alzheimer’s disease is a common, progressive and irreversible neurodegenerative disease, contributing to 60–80% of all cases of dementia worldwide [113]. The typical pathological hallmarks of AD consist of deposits of amyloid β-peptide (Aβ) plaques, neurofibrillary tangles (NFTs) with hyperphosphorylated tau and neurodegeneration [113]. In addition, the BBB dysfunction has been implied as a putative upstream mechanism of AD pathology independent of Aβ and tau, offering pathways for peripheral immune cells to traffic into the brain and involve in disease progression [114].

Prior research shows no discrepancy in the quantity and proportion of NK cells in PBMCs between different stages of dementia and healthy controls [115–117]. Instead, bioinformatics analyses conclude a significant decrease of NK cells in the peripheral blood of AD patients at single-cell resolution [118, 119]. As for functional alterations, enhanced cytotoxicity and stronger cytokine production of peripheral NK cells are detected in patients with amnestic mild cognitive impairment (aMCI), but there is a reduced cytotoxic activity in patients with later stage of AD, suggesting that NK cells may response actively in the early stage but lose function during disease progression [117, 119]. In 7–8 months 3xTg-AD model, NK cells mainly resident in the border of CNS, like leptomeninges, choroid plexus and perivascular space, and skew to a proinflammatory profiles. Blocking the action of NK cells with anti-NK1.1 antibody, researchers found no detectable changes in amyloid Aβ deposition but amelioration of microglial activation and improvement in neurogenesis and cognitive function [120]. Of note, NK cells seem to interact with neural stem cells in the SVZ regions, similar to what has been observed in multiple sclerosis [65]. Due to limited studies, whether or not NK cells contribute to the pathogenesis of AD and their precise role in different stage of the disease remain unclear and needs to be further investigation.

DAP12 (also known as TYROBP), a signal transduction element mainly expressed by NK cells and myeloid cells, act as an adaptor to transmit signals received from immune receptors [121]. DAP12 has been linked to cognitive function, as loss of functional DAP12 resulting in Nasu-Hakola disease (NHD) characterized by multiple bone cysts and presenile neurodegeneration [122]. Moreover, DAP12 is also regarded as a crucial genetic factor in late-onset Alzheimer’s disease [123]. DAP12 expressed by microglia regulates amyloid burden and tau protein phosphorylation [124, 125]. Of note, DAP12 is essential for NK cell cytotoxicity and cytokine secretion [126]. One case report describes NHD patients harboring TYROBP mutations exhibits decreased number of NK cells and immune dysfunction, similar to phenotypes of Tyrobp KO mice, suggesting NK cells may be involved in DAP12-mediated cognitive alterations in dementia [127]. Further work is required to address the link between NK cells and dementia and its related questions.

##### Parkinson’s disease

Parkinson’s disease is a progressive neurodegenerative disorder that mainly affects substantia nigra, causing impaired movement. Abnormal α-synuclein aggregates and composes Lewy bodies, the pathological hallmark of PD, which can activate both innate and adaptive immunity [128]. Assessment of PBMCs indicates that dysregulation of NK cell response might participate in PD. For example, a decreased CD57^+^CD28^+^ NK cell subtype, associated with a more terminal and active phenotype of NK cells, is observed in patients with PD [129]. Migration of NK cells to the brain might be impaired owing to downregulation of the adhesion molecule VLA-4 on the surface of patients’ peripheral blood NK cells [31, 130]. NK cells are present in the substantia nigra of PD patients [131]. Like other innate immune cells, they also express Toll-like receptors (TLRs), a key cluster of pattern recognition receptors (PRRs) to recognize pathogen-associated molecular patterns (PAMP) and damage-associated molecular patterns (DAMP), which makes them efficiently uptake and degrade extracellular α-synuclein by TLR2 and TLR4 in the absence of excessive self-activation [132]. Deficiency of NK cells in a PD model results in exacerbated synuclein pathology, glial cells activation and death of dopaminergic neuron, which suggests NK cells can be a potential scavenger to abnormal proteins (Fig. 2) [131]. A recent study analyzed single-cell RNA sequencing data and found the ratio of NK cells increased in PD patients’ CSF, especially a neuroprotective RAC1^+^ subset associated with substantia nigra development [133]. Based on these findings, targeting NK cells might be a potential therapeutic strategy for PD treatment. Evidence on the involvement of autoimmunity in PD pathology mechanisms continues to accumulate, as α-synuclein can be a strong autoantigen to initiate adaptive immune [128]. Given the regulatory capacity of NK cells in the adaptive immunity, more research is warranted for exploration of NK cell functions in regulating adaptive immune responses in PD.

##### Amyotrophic lateral sclerosis

Amyotrophic lateral sclerosis (ALS), also known as motor neuron disease (MND), is a progressive neurodegenerative disease that mainly damage motor neurons [134]. Comparing NK cells percentage from the blood samples of ALS patients and healthy controls, different studies showed diverse results, ranging from increased to deceased NK cells in the diseased state [40, 135, 136]. Recent research reports the circulating NK cell number is unchanged but there is a significant increase in some markers involved in NK cell cytotoxicity and trafficking, including NKG2D, NKp46, CD11a, CD11b, CD38, and CX3CR1, and all of them are correlate with ALS severity [137]. In post mortem tissue of patients with ALS, NK cells are found to extend into the spinal cord and cerebral motor cortex, which is also confirmed in the hSOD1^G93A^ mice, an ALS transgenic model. They infiltrate to the injured areas via CCL2 secreted by neurons and ablation of NK cells delays the onset of the disease. After trafficking to the CNS, NK cells exert the cytolytic function to damage motor neurons and produce IFN-γ to drive an inflammatory microglia phenotype and limit the entrance of Treg, all of which accelerate the neuroinflammation and neurodegeneration of ALS (Fig. 2) [40]. Based on the above findings, researchers attempt to apply tofacitinib, an FDA-approved immunomodulating medication for autoimmune diseases, to ALS. They found tofacitinib successfully inhibited NK cell functions and decreased the cytotoxicity to ALS-patient derived neurons in vitro. Although the experiment does not provide the in vivo data about therapeutic effects to neurologic deficits, targeting NK cells might be a promising strategy to treat neurodegeneration [138].

#### Cerebrovascular diseases

Ischemic stroke, a prevalent cerebrovascular disease, is frequently accompanied by the infiltration of peripheral immune cells. Unlike the etiology of MS, the abrupt cessation of blood flow in ischemic stroke causes endothelial cells to express elevated levels of adhesion molecules and induces subsequent damage of parenchymal cells, particularly neurons, which then release DAMPs. Consequently, brain-resident immune cells like microglia are activated and secrete cytokines and chemokines, potentially promoting peripheral leukocyte adhesion, chemotaxis, and extravasation into the parenchyma [139]. Notably, the post-stroke immune cell infiltration displays temporal dynamics, characterized by innate immune cell infiltration in the early stage (typically within 24 h post-onslaught) and a subsequent adaptive immune cell response usually between days 3 to 7 [140–143]. Although NK cells constitute only a small proportion of infiltrating lymphocytes, they may play a crucial role in initiating and amplifying post-stroke inflammation and injury [140]. Increasing NK cells are found in the infarct and periinfarct brain regions of both patients and middle cerebral artery occlusion (MCAO) mouse models, which are chemoattracted via CX3CL1 produced by damaged neurons and IP-10 secreted by glial cells and endothelial cells [37, 41]. IL-15 is upregulated in astrocytes after ischemic stroke and augments NK cell priming and activation, blockade of which by antibodies or gene knockout ameliorates ischemic brain injury [21, 144]. NK cells activation and cytotoxicity after infiltration is associated with the loss of the self-tolerant molecule Qa1 of hypoxic neuron and the upregulation of activation receptor NKG2D on NK cells [37]. Regarding their effector functions, NK cells can kill ischemic neurons either directly through secreting perforin or indirectly releasing IFN-γ. The latter can not only induce neuronal excitotoxicity but also polarize the phenotype of microglia and macrophages to exacerbate neuroinflammation (Fig. 2) [37, 145]. An experiment using an in vitro BBB model after oxygen glucose deprivation (OGD) treatment suggests that NK cells’ involvement in the BBB impairment as well [41]. Consequently, NK cells could potentially play a pivotal role during stroke onset, and inhibiting NK cells at early stage may help disrupt the neuroinflammation feedback loop therefore carry therapeutic potential for the disease.

Hemorrhagic stroke can be divided into intracerebral hemorrhage (ICH) and subarachnoid hemorrhage (SAH). NK cells, which invade into the brain regions adjacent to the lesion after ICH in both patients and mouse models, mediate the BBB damage and resulting brain edema. Specifically, brain endothelial cells after ICH undergo the downregulation of MHC-I molecule H2-Kb and enhanced expression of NK-activating ligands RAE1 and MULT-1. Apart from direct damage to the BBB, infiltrated NK cells also assist in recruiting neutrophils by producing CXCL2, exacerbating perihematomal edema (Fig. 2) [146, 147]. The elevated counts of activated CD56^dim^ NK cells are observed in the CSF of individuals with SAH as well, indicating a possible cytotoxic role of NK cells in the pathology of SAH [148, 149].

Cerebral small vessel disease (CSVD) is a chronic and progressive disease related to the small blood vessels in the brain. A recent study found cytotoxic CD56^dim^ NK cells increased and correlated with sparse nerve fibers in Aging-related atherosclerotic CSVD patients. Based on proteomic analyses, researchers found CD56^dim^ NK cells contribute to the BBB breakdown via the secretion of cathepsin D (CTSD). Furthermore, these cells infiltrate into the parenchyma and disrupt nerve fibers by releasing granzyme H (GZMH) [150]. These findings suggest NK cells may drive neural injury, exacerbate inflammation, and disrupt the BBB. Blocking the action of NK cells may be a promising treatment to improve outcome in both acute and chronic cerebrovascular diseases.

#### Infection

NK cells are best known to help the body fight infection at an early stage and provide a first line of defense against pathogens invading the CNS, especially intracellular pathogens (viruses, bacteria, and parasites) [151]. Once infection occurs, peripheral NK cells migrate to the affected sites accompanied with trNK cell activation [152, 153]. However, the balance between the resistance to infection and the undesired tissue damage and immunopathology determines the disease outcomes [9]. For example, IFN-γ produced by NK cells and ILC1s limits the proliferation of granuleneuron progenitor cells (GNPCs) and induces abnormal cerebellar development after cytomegalovirus (CMV) infection (Fig. 2) [43, 154]. Additionally, NK cells are found in the brain with enhanced cytotoxicity and secretory abilities in an A. cantonensis-infected mice model, another parasite affecting the CNS, suggesting they are an accomplice of pathogens. In contrast, cerebral malaria is the most life-threatening neurological complication of Plasmodium falciparum infection with characterized by microvasculature malfunction and dysregulated inflammation [155, 156]. Based on the potentially immunoregulatory role of NK cells and apparently incongruous immune responses in ECM, IL-15 treatment in ECM successfully prevent the onset of the brain injury without altering the parasite load by inducing NK cell persistent STAT3 activation and secretion of anti-inflammatory IL-10 [157, 158]. IL-10 derived from NK cells attenuates the activation of CD8^+^ T cells, mitigates disintegration of the BBB, and ameliorates brain edema, thereby improving the survival rates greatly (Fig. 2).

### Therapeutic potential and future perspectives

Activated NK cells can induce cell cytotoxicity, produce multiple cytokines and drive a pro-inflammatory environment, resulting in brain injuries. On the contrary, NK cells are able to clear out abnormal protein aggregation, regulate the inflammatory milieu and alleviate the brain damage caused by overreacting immune responses. Therefore, precise manipulation of NK cells can be promising therapeutic strategies to cope with different neurological diseases: inhibition of NK cell trafficking, activation, and effector functions may reduce NK-mediated brain damage, while restoration of dysregulated NK cell functions may limit harmful neuroinflammation. There are several existing treatments involved in enhancement of NK cell functions to balance abnormal neuroinflammation in MS as discussed above. More strategies can be learnt from cancer therapies. For instance, immunomodulatory agents, like thalidomide and related drugs, cytokines, like IL-15, antibodies blocking inhibitory receptors, and adoptive transferring of NK cells might become effective ways to restore NK cell functions. Targeting specific NK-activating or inhibitory signals might achieve goals of protecting “wanted” cells and eliminating “unwanted” cells dependently on different situations.

Chimeric antigen receptors (CARs) are a type of receptor proteins that endows immune cells with abilities to target specific antigenic proteins [159]. CAR-NK cells equipped with a wide range of tumor antigens have been advanced into preclinical trials for the treatment of different cancers [160]. In addition to cancer therapy, transferring anti-CD19 CAR-T cells successfully ameliorates CNS autoimmunity via B cell depletion in EAE mice [161]. Similarly, engineered CAR-NK cells with specific antigens are potential strategies to eliminate target cells, like autoreactive T cells and overactivated microglia to promote recovery. In addition, without modifying to express CARs, off-the-shelf NK cell lines or induced pluripotent stem cell-derived NK (iPSC-NK) cells show enhanced functional potential, transplantation of which might restore the functional deficiency of NK cells, especially in MS, and inhibit excessive immune responses [162]. Besides, transplantation of extracellular vesicles (EVs) loaded with various proteins, lipids, and nucleic acids have been considered as a viable alternative of cell therapy because of better convenience and higher safety [163]. The NK cell-derived EVs have been shown to contain components such as granzymes, perforin and FSAL, which showed cytotoxic effects on tumor cells in both cultured cells and animal models without significant side effects on normal cells [164–166]. Recent research showed NK cell-derived exosomes also alleviated depression in mice by controlling the inflammation of astrocytes via transferring miRNAs, indicating the immunotherapeutic potential to the neurological diseases [167].

Although achieving much progress, there are still many unknowns. Firstly, finding out whether tissue-resident NK cells exist in the human CNS and their specific markers is obviously important. Decidual natural killer (dNK) cells are the critical type of trNK cells supporting maternal–fetal tolerance. The maternal–fetal interface resembles the BCSFB, serving as an active but controlled gate rather than an inert, impermeable barrier [168]. dNK cells regulate the immune microenvironment by inducing Tregs and inhibiting effector T cells during early pregnancy to ensure immunological endurance [169]. Similarly, as CD56^bright^ NK cells and ILC1s are enriched in the choroid plexus, they might play an important role as a gatekeeper to the BCSFB under normal conditions. Once the immunoregulatory capacity of NK cells is impaired, it might initiate uncontrolled inflammatory reactions and cause diseases in the CNS. Due to potential and crucial functions of NK cells, it is therefore important to develop genetic tools, such as transgenic mouse models, to help study and distinguish the different roles between brain-resident and circulating NK cells. Secondly, whether NK cells involved in maintaining normal brain functions in addition to immune functions remains to be determined, since increasing evidence suggests that immune cells may support cognitive functions of the brain [170]. Taking dNK cells as example, it is shown that activated dNK cells release the cytokines and chemokines that mediate trophoblast invasion, angiogenesis and uterine artery remodeling, which plays vital roles in placental and embryo development [169]. It is also critical to explore the interaction between NK cells and intrinsic cells of the brain and clarify their communication signals as the suspected role of NK cells regulating in normal brain functions. Besides, because many neurological diseases are co-participated by CNS-resident cells and other peripheral immune cells, to what extent do NK cells contribute to the development of these diseases? Are they play a direct or supporting role in neuroinflammation? In addition, it has been appreciated that human NK cells exhibit great heterogeneity with distinct distribution, phenotypic and functional features. Do specific subsets of NK cells exist that correspond to different neurological diseases? It may also carry potential therapeutic advantage in identifying the ways to target certain subpopulation of NK cells which is involved in the CNS diseases.

## Conclusions

Various studies demonstrate that NK cells interact with the CNS extensively under different circumstances. Despite their relatively small proportion within the CNS, NK cells may act as both conciliators and instigators in neuroinflammation, which underlines their potential for coordinating the immune system and the CNS. However, their exact roles related to the health and disorders, as well as the mechanisms for their accurate regulation still remain unclear. Through more in-depth exploration, we can understand the communications between NK cells and the CNS better, thereby providing evidence for treatments.

## Data Availability

Not applicable.

## Abbreviations

AD: Alzheimer’s disease
AEs: Adverse events
ADCC: Antibody-dependent cellular cytotoxicity
ALS: Amyotrophic lateral sclerosis
BBB: Blood–brain barrier
BCSFB: Blood-cerebrospinal fluid barrier
CARs: Chimeric antigen receptors
ChAT: Choline acetyltransferase
CLP: Common lymphocyte progenitor
CMV: Cytomegalovirus
cNK: Conventional NK
CNS: Central nervous system
CSF: Cerebrospinal fluid
CSVD: Cerebral small vessel disease
CTSD: Cathepsin D
DAMP: Damage-associated molecular patterns
dCLNs: Deep cervical lymph nodes
DG: Dentate gyrus
dNK: Decidual NK
EAE: Experimental autoimmune encephalomyelitis
EVs: Extracellular vesicles
FASL: FAS ligand
GM-CSF: Granulocyte–macrophage colony-stimulating factor
GNPCs: Granuleneuron progenitor cells
Gr: Transferring granzyme
GZMH: Granzyme H
HSCT: Hematopoietic stem cell transplantation
ICH: Intracerebral hemorrhage
IFN-β: Interferon β
ILC: Innate lymphoid cell
ILC1s: Type 1 innate lymphoid cells
iPSC: Induced pluripotent stem cell
ITAMs: Immunoreceptor tyrosine-based activation motifs
LTi cells: Lymphoid tissue inducer cells
MCAO: Middle cerebral artery occlusion
MS: Multiple Sclerosis
NHD: Nasu-Hakola disease
NK cells: Natural killer cells
NKRs: NK cell receptors
NSCs: Neural stem cells
OGD: Oxygen glucose deprivation
PAMP: Pathogen-associated molecular patterns
PD: Parkinson’s disease
PRRs: Pattern recognition receptors
RRMS: Relapsing remitting MS
SAH: Subarachnoid hemorrhage
SASP: Senescence-associated secretory phenotype
sCLNs: Superficial cervical lymph nodes
SGZ: Subgranular zone
SVZ: Subventricular zone
TLRs: Toll-like receptors
TNF: Tumor necrosis factor
TRAIL: TNF-related apoptosis-inducing ligand
trNK: Tissue-resident NK
